# Association mapping for yield and grain quality traits in rice (*Oryza sativa* L.)

**DOI:** 10.1590/S1415-47572010005000065

**Published:** 2010-09-01

**Authors:** Tereza Cristina de Oliveira Borba, Rosana Pereira Vianello Brondani, Flávio Breseghello, Alexandre Siqueira Guedes Coelho, João Antônio Mendonça, Paulo Hideo Nakano Rangel, Claudio Brondani

**Affiliations:** 1 Embrapa Arroz e Feijão, Santo Antônio de Goiás, GOBrazil; 2 Setor de Melhoramento de Plantas, Escola de Agronomia, Campus-II Samambaia, Universidade Federal de Goiás, Goiânia, GOBrazil

**Keywords:** association analysis, core collection, genetic structure

## Abstract

Association analysis was applied to a panel of accessions of Embrapa Rice Core Collection (ERiCC) with 86 SSR and field data from two experiments. A clear subdivision between lowland and upland accessions was apparent, thereby indicating the presence of population structure. Thirty-two accessions with admixed ancestry were identified through structure analysis, these being discarded from association analysis, thus leaving 210 accessions subdivided into two panels. The association of yield and grain-quality traits with SSR was undertaken with a mixed linear model, with markers and subpopulation as fixed factors, and kinship matrix as a random factor. Eight markers from the two appraised panels showed significant association with four different traits, although only one (RM190) maintained the marker-trait association across years and cultivation. The significant association detected between amylose content and RM190 was in agreement with previous QTL analyses in the literature. Herein, the feasibility of undertaking association analysis in conjunction with germplasm characterization was demonstrated, even when considering low marker density. The high linkage disequilibrium expected in rice lines and cultivars facilitates the detection of marker-trait associations for implementing marker assisted selection, and the mining of alleles related to important traits in germplasm.

## Introduction

Association analysis, or linkage disequilibrium mapping, is a notable strategy used for identifying genes controlling important traits. It is already being successfully applied for identifying genes related to human diseases. Research in humans has turned to association analysis, since linkage analysis has not been successful in the fine-scale mapping of disease loci, due to the impossibility of undertaking controlled-breeding crosses (Flint-Garcia *et al.*, 2003). Unlike humans, in most plant species, the identification of those genomic regions which contribute to important characteristics has been mostly achieved through linkage analysis within segregating populations, the result of crosses between genitors with contrasting phenotypes and genotypes (Buntjer *et al.*, 2005; Skot *et al.*, 2005).

In breeding and pre-breeding programs, QTL detection is an important tool for the identification of favorable alleles and for identifying and validating molecular assisted selection (MAS). The identification of genomic regions related to quantitative traits in plants was largely achieved through QTL mapping (Skot *et al.*, 2005). However, there are some inherent limitations to QTL analysis. First, in linkage studies, the segregating population usually presents only two segregating alleles per locus, which means that in any given cross, the sampled genetic diversity may be limited. In addition, the potential use of the genetic diversity available in species is restricted (Peleman and van der Voort, 2003). Another disadvantage as regards the substantiation of a low number of traits per cross, is through the difficulty in identifying parents with contrasting genotypes and phenotypes for all those traits of interest (Buntjer *et al.*, 2005). Furthermore, the high resolution desired for MAS or cloning candidate genes requires developing large segregating populations, possibly difficult in some species (Skot *et al.*, 2005).

According to Zondervan and Cardon (2004), the main purpose in linkage analysis, as in association mapping, is the detection of correlations between phenotypic variation and genotypes through linkage disequilibrium. However, association analysis has the advantage of contemplating all the meiotic and recombination events that may occur in the evaluated population (Ferreira and Grattapaglia, 2006). Furthermore, this form is highly dependent on the extent of linkage disequilibrium (LD), a higher degree implying the use of less markers per chromosome, without the loss of genetic resolution for marker assisted selection (MAS) (Rostoks *et al.*, 2006). As rice is a self-pollinating species, it is expected to present high linkage disequilibrium (Flint-Garcia *et al.*, 2003), thereby requiring fewer markers. In addition, the recent bottlenecks encountered since the beginning of rice breeding have given rise to high linkage disequilibrium blocks, thereby facilitating association studies (Patron *et al.*, 2002).

One of the great advantages of association mapping lies in the fact that no mapping population needs to be developed, as the sampling of non-related individuals represents a series of advantages towards developing and validating MAS in breeding programs (Jannink *et al.*, 2001), as well as an opportunity for increasing the exploitation of germplasm accessions in the search for advantageous allele combinations. Such a strategy, unlike traditional linkage analysis, facilitates the search for functional variation in a much broader germplasm context (Zhu *et al.*, 2008). Thus, experimental populations may constitute a representative sample of a larger population for which inferences are sought (Breseghello and Sorrels, 2006b). In panels with highly divergent individuals and assumed random mating, only polymorphisms with extremely tight linkage to a locus with desirable phenotypic effects are likely to be significantly associated with a given trait (Remington *et al.*, 2001). Furthermore, association analysis can benefit by including data collected over years of experimental analysis with genotypes of breeding programs, with the additional possibility of analyzing several traits simultaneously.

Improving grain yield and quality are important challenges in rice breeding, thus priorities for the international market (Fan *et al.*, 2005). Although quality assumes many aspects and is highly related to preference in diverse cultures, its characteristics are mainly defined by milling properties, grain size and shape, cooking and eating characteristics, and nutritional qualities (He *et al.*, 1999). According to He *et al. (*1999), of these the most relevant are appearance and cooking quality, reported to be directly related to amylose content, gel consistency and gelatinization temperature (Fan *et al.*, 2005).

The aims of this work were to analyze and identify the association of simple sequence repeat (SSR) markers with yield and grain quality traits in a panel of accessions from the Embrapa Rice Core Collection (ERiCC), represented by breeding material from Brazil and other countries.

## Material and Methods

###  Plant material and genomic DNA extraction

The evaluated panel of 242 accessions from ERiCC (Abadie *et al.*, 2005) was composed of: a) 94 accessions of inbred lines and cultivars developed by rice breeding programs in Brazil (57 upland and 37 lowland accessions); and b) 148 accessions of inbred lines and cultivars developed by breeding programs worldwide (76 upland and 72 lowland accessions) (Table S1). Each accession was evaluated in a four-plant bulk, the total genomic DNA being extracted as described by Brondani *et al.* (2002).

**vPhenotypic data**

The phenotypic evaluation of 242 ERiCC accessions was carried out in Santo Antônio de Goiás, the state of Goiás, Brazil (altitude 749 m; 16°40'43” S; 49°15'14” W), in 2004 and 2005, under irrigated conditions, following an augmented block design with plots of 4 rows x 5 m, at a density of 20 plants m^-1^. Data were taken from the two middle rows and the 4 central meters of each. In 2004, the following traits were evaluated: 1) YLD - grain yield, in kg ha^-1^; 2) TILN - tiller number per plant; and 3) PANN - panicle number per plant. In 2005, two traits were evaluated: YLD and the yield from ratooning (RYLD), this being the yield from the second harvest, approximately 40 days post-main. In both years, grain quality data were evaluated for cooking and milling traits, measured as to amylose content (AC) and head-milled rice (MR), respectively. Amylose content was determined according to Juliano (1979), whereas head-milled rice was measured as the proportion of the weight of whole kernels over the weight of paddy rice. The descriptive statistics of phenotypic data were computed using the Genes 4.1 program (Cruz, 1997).

###  SSR characterization of ERiCC

The 86 SSR fluorescent markers were dispersed in all the 12 rice chromosomes, an average of seven markers per chromosome (ranging from a maximum of eight markers and a minimum of five per chromosome). The markers were labeled with the fluorescent dyes HEX (hexachlorine - 6 carboxyfluorescein) and 6-FAM (fluorochrome 6-carboxyfluorescein) (Table S2). PCR was carried out in a final volume of 15 μL containing a customized concentration of primers (forward and reverse) (Table S2), 1X reaction buffer (50 mM of KCl, 10 mM Tris-HCl pH 8.4, 0.1% Triton X-100 and 1.5 mM of MgCl2), 0.22 mM of dNTP, 15 ng of template DNA, and one unit of the Taq DNA Polymerase enzyme. Thermocycling was carried out in a GeneAmp PCR System 9700 (Applied Biosystems) and the amplification conditions were 94 °C for 5 min followed by 40 cycles of 94 °C for 1 min, specific annealing temperature for 1 min, and 72 °C for 1 min, and a final extension of 72 °C for 7 min. PCR products were analyzed in an ABI 3100 DNA sequencer (Applied Biosystems) and the alleles were scored with GeneMapper 3.5 software (Applied Biosystems). The size standard used was obtained according to Brondani and Grattapaglia (2001).

###  Statistical analysis

*Allele diversity and genetic structure:* The 242 inbred lines and cultivars of ERiCC were analyzed in a pooled DNA sample, composed of four individual plants. From SSR analysis, heterogeneity (the presence of individual heterozygous or homozygous plants with different alleles in the bulk) was evident in certain accessions. In order to proceed with association analysis, the accessions were treated as pure lines, under the definition of working alleles, and represented by the most common allele. Rare alleles (frequency below 5%) were treated as missing data in population structure analysis, and as null alleles in association analysis, according to the strategy described by Breseghello and Sorrels (2006a). The hypothesis of division from one to four subpopulations was tested with structure software (Pritchard *et al.*, 2000), allowing for admixture and correlated allele frequencies, with a burn-in of 10,000 and a run-length of 100,000. The *Fst* parameter (software FSTAT 2.9.3.2; Goudet, 2002) and factorial correspondence analysis (FCA) (software Genetix 4.03; Belkhir *et al.*, 2004) were also applied for investigating accession genetic structure.

*Association analysis:* The association between markers and phenotypic traits was done using the Mixed Linear Model (MLM), an available option in Tassel version 1.9.6 software, where markers tested and subpopulation data (Q matrix) were considered as fixed-effect factors, whereas the kinship matrix was considered as a random-effect factor. The kinship matrix was obtained from SPAGeDi version 1.2 software (Hardy and Vekemans, 2002). To confirm the significance of associations between loci and traits, a correction for multiple testing was applied using the false discovery rate (FDR) method with Qvalue version 1.0 software (Storey, 2002). The FDR level was set at 0.05, and the π_0_ method for bootstrap analysis. The FDR method, expressed as a *q*-value, is defined as the expected proportion of true null hypotheses within the class of rejected null hypotheses (Kraakman *et al.*, 2004).

The significance of differences between allele effects was obtained from Kruskal-Wallis nonparametric rank testing with *R* program (*R* Development Core Team). Non-parametrical multiple test procedures, regarding amylose content and each pair of alleles, were carried out as described by Campos (1983).

## Results

###  Phenotypic data

Experimental field data were distributed normally, except for traits related to grain quality (AC and MR). ERiCC accessions revealed wide variation in performance of all the evaluated traits (Table 1). In the 2004 experiment, the firmest correlation detected was between TILN and PANN (0.88; p < 0.01), and in the 2005, between YLD and MR (0.38; p < 0.01) (Table 2). No correlation was detected in yield data from 2004 and 2005 experiments.

###  Allele diversity and population structure

A total of 1,066 alleles were detected with the set of 86 SSR markers on a panel of 242 accessions. The average number of alleles per locus was 12.4, ranging from three (RM484) to 32 (RM204). Most loci presented one or more alleles with a frequency below 5%. These rare alleles represented, approximately, 48% of the total allele number, and to avoid an increase in variance errors in association analysis, they were not considered. The remaining alleles (554), referred to as common alleles, ranged from two to eight per locus. In lowland accessions, the mean was 4.80 alleles per locus, with gene diversity of 0.64, whereas in upland accessions this was 4.76 alleles per locus and gene diversity 0.56. Common SSR alleles were used to check the structure of ERiCC genetic variation. The model-based clustering method resulted in the highest likelihood from data, *i.e.* the probability that a given individual originated from a certain population, when the number of subpopulations (k) was set at 2, thereby indicating a subdivision among accessions caused by the cultivation system (lowland or upland rice accessions). No population structure was detected due to the origin of accessions (Brazilian or worldwide breeding programs).

**Figure 1:**
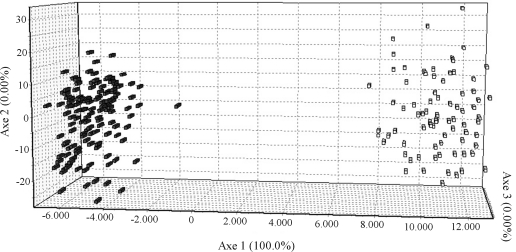
Spatial distribution of genetic variability in the 210 selected accessions from ERiCC, based on factorial correspondence analysis (FCA). The white dots represent lowland accessions and the gray, uplandones.

Thirty-two accessions (13%) were predicted to have admixed ancestry, this meaning that their origin could not be attributed exclusively to one of the two inferred subpopulations. Consequently, they were discarded from the analysis. Association analysis was then undertaken with 210 ERiCC accessions (92 lowland and 118 upland). The overall *Fst* statistics across subpopulations was 0.775, and *Fst* values for lowland and upland groups were 0.135 and 0.205, respectively, thereby indicating high differentiation among subpopulations, and low to intermediate levels of differentiation within accessions from the same subpopulation. FCA was applied in order to visualize the subdivision among accessions, whereby it was possible to confirm the division into two subpopulations, based on the cultivation system (Figure 1).

###  Association analysis

Three different accession panels, viz., a complete panel of 210 accessions, and from this, 92 lowland accessions and 118 upland, were analyzed (association analysis). 23 SSR markers (27%) in the complete panel were identified as being significantly associated to at least one of the evaluated traits (data not shown). On considering the low genomic coverage of the SSR set, and the complexity of the traits evaluated, the number of associated markers was high, thereby implying the presence of spurious marker-trait associations. On considering the system of cultivation as the main generator of genetic structuring among all the 210 accessions, and using subpopulations as a basis for re-analysis, two accession panels were defined, with a lower number of marker-trait associations. From the 86 SSRs, eight markers were associated with four traits in lowland, and only one with amylose content in upland accessions (Table 3). In lowland accessions, the marker OG60 was associated with PANN in the 2004 experiment and RM190 with AC in both the 2004 and 2005. In 2005, two markers (RM1 and 4653) were associated with MR, and four (RM264, RM267, RM125 and RM38) with YLD. In upland accessions and in both years, there was a significant association of only RM190 with AC. There was no association with any marker in the case of RYLD and TILN. Despite the significant associations between marker-trait, the only consistent association over years and cropping systems was between the RM190 marker and AC. This association was also significant in the analysis of all the 210 accessions (data not shown).

**Figure 2:**
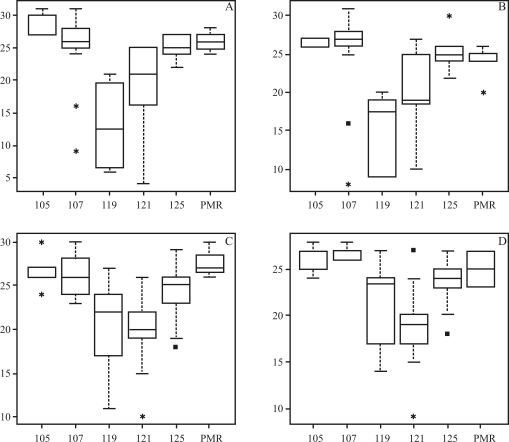
Empirical distribution of amylose content (*y*-axis) among alleles identified for RM190 SSR (*x*-axis). The subdivisions in amylose content data refer to quartile division, and the lines in boxes are the median of amylose content in each allele. The A and B boxes refer to amylose content data on lowland accessions from the 2004 and 2005 experiments, respectively. The C and D boxes refer to amylose content data on upland accessions from the 2004 and 2005 experiments, respectively. The pool of rare and missing alleles is represented by the PMR denominated allele.

On considering the 210 accessions, six alleles (frequencies over 5%) were distinguished for RM190, with four common in both accession panels (alleles 105, 107, 121 and 125 bp). However, two alleles (117 and 119 bp) were identified (frequencies over 5%) one each only in upland and lowland accession panels, respectively (Figure 2). From Kruskal-Wallis testing, it was possible to identify a significant difference in allele effects across lowland and upland panel accessions From a multiple test based on a non-parametrical approach it was possible to identify the relationships of the six RM190 alleles with diverse classes of amylose content, as well as positive and significant correlation, for both years, in upland and lowland panel accessions (0.58 with p < 0.01, and 0.54 with p < 0.01, respectively).

In the lowland accession panel, the 105 and 107 bp (base pair) alleles presented significantly different effects from the remainder. According to 2004 and 2005 experimental data, both were correlated to higher amylose content than the others, although in 2004, there was a significant difference between the two themselves, with the 105 bp revealing higher AC than the 107 bp (Table 4). Despite statistical differences between AC and the respective alleles, no clear pattern in allele effects could be identified in lowland accessions. In the upland accession panel, both in 2004 and 2005, 121 bp allele AC was lower than in 105, 107 and 125 bp, whereas in 2005, 117 bp allele AC was lower than in 105 and 107 bp. As with lowland accessions, no clear pattern in allele effects was identified.

## Discussion

###  SSR allele diversity and population structure

A set of 86 highly informative SSR markers was used in genotyping ERiCC inbred lines and cultivars. The number of common alleles detected (frequency ≥ 5%) was similar to that previously identified for inbred rice lines and cultivars (Lu *et al.*, 2005). In the present work, rare alleles were not integrated into analysis, as low frequency alleles inflate variance estimates of linkage disequilibrium (Remington *et al.*, 2001). Additionally, rare alleles are more susceptible to bias caused by covariance between markers and population structure, thus increasing the chance of type I error (Breseghello and Sorrels, 2006a). The presence of admixture also contributes to overestimating linkage disequilibrium, due to causes not related to physical connections on a chromosome (Flint-Garcia *et al.*, 2003).

According to Breseghello and Sorrels (2006a), the selection process of a minimum sample with maximum variation results in a normalizing effect that is expected to minimize population structure, thereby creating a favorable situation for association analysis. Structure-presence specification is a prior requirement in core collections, since certain procedures adopted to build these collections may lead to a structured accession panel. The stratification of accessions into meaningful groups, with the maximum variation between groups and the minimum within, as suggested by van Hintum *et al.* (2000), may result in accession panels with low potential for detecting gene effects through association analysis, since most variance is attributed to population structure. ERiCC inbred lines and cultivars were originally selected according to the origin of accessions (Brazil or worldwide breeding programs), and their system of cultivation (lowland or upland) (Abadie *et al.*, 2005). The ERiCC structure based on a cultivation system is probably due to most lowland accessions being from *indica* subspecies, whereas upland accessions are mostly from *japonica* (Khush, 1997).

A very substantial differentiation between lowland and upland accessions was identified, after the removal of rare alleles and admixed accessions, with *Fst* increasing from 0.11 to 0.77. In Garris *et al.* (2005), *Fst* values for *indica* and *temperate japonica* and *indica* and *tropical japonica* accessions were, respectively, 0.43 and 0.36, with the proportion of admixed ancestry estimated at 10%. The higher *Fst* value identified between lowland and upland accession panels, even when compared to the divergent *indica* and *japonica* accessions found by Garris *et al.* (2005), may be due to the elimination of rare alleles and accessions with mixed ancestry, thereby increasing divergence between the two.

###  Association analysis

Despite the broad genome coverage provided by the set of 86 SSR markers used in the analysis, the whole genome scan designed for association analysis was limited by the low density of markers. However, due to numerous bottlenecks in the history of rice domestication, the level of linkage disequilibrium in some regions is estimated to be greater than the 250 kb found in *Arabidopsis* (Garris *et al.*, 2003). Considering this situation, a target marker can be associated to a candidate gene responsible for certain traits, even though thousands of base pairs apart, thereby favoring marker-assisted selection procedures.

Association analysis with MLM combines information from kinship and population structure, thereby reducing type I errors (Yu *et al.*, 2006), that is the error of rejecting a null hypothesis when it is actually true. Here, 23 SSR markers, associated to yield and grain quality traits, were identified, when such an approach was applied to the panel of 210 accessions. Agrama *et al.* (2007), on using the same model in association analysis of yields and their components in 103 rice germplasm accessions with 123 SSR markers, identified 25 marker-trait associations with 21 SSRs. The main subpopulations identified from their data set corresponded to the geographic origin of accessions. However, from our results there was every indication that the subdivision of the evaluated accessions could be attributed to differences in the cultivation system. In the case of Agrama *et al.* (2007), the difference could be due to bias in favor of *japonica* as against *indica* accessions, for, from the 103 rice accessions, 49 corresponded to japonica accessions, whereas 25 were classified as of an admixed ancestry, mainly between those with an *indica* and temperate *japonica* background.

In a comparative analysis of yield components, with data from 2004 and 2005, a difference was noted, from one year to the next, in the marker-trait association of yield-related genes, as detected by SSR markers. Quantitative agronomic traits, such as yield, are especially affected by the interaction of both genotype and environment, an understanding of how this interaction is controlled being a basis for defining breeding strategies that would improve genetic gains within these traits.

Two markers (RM38 and RM267) were previously detected by QTL mapping. RM38 was related to yield when assaying 190 lines in an *indica* x j*aponica* double-haploid population, genotyped with 179 SSR markers for agronomic traits in a two-year replicated-field experiment (Jiang *et al.*, 2004). This finding was corroborated by Marri *et al.* (2005) who also found the RM38 marker related to a yield QTL (R^2^ of 7.99), by using an interspecific population of 251 advanced backcross families (*O. rufipogon* x *O. sativa*), genotyped with 80 SSR markers. Cho *et al.* (2003) identified the association of RM267 with a yield QTL detected in an interspecific backcross F_2_ population (*O. rufipogon* x *O. sativa*). A recent study in rice, dealt with the association between markers previously identified as linked at QTLs (Agrama *et al.*, 2007). With this base, QTL analysis can be considered a pre-requisite for distinguishing markers related to important traits, by constituting, together with complementary association studies using highly divergent accession panels from core collections, a means of identifying the different alleles of these markers and attributing phenotypic weights to each.

No difference in allelic effects on yield was identified through association analysis, when considering markers previously associated to this trait in rice. This may be due to the complex inheritance of the YLD trait, as not only a single allele, but also epistatic alleles are involved. Haplotypes related to highest performance may be identified through association analysis, and, an additional advantage over QTL mapping analysis, values can be attributed to alleles present in a given panel of individuals, whereby the most favorable combinations can be traced by breeders in all elite lines and cultivars. Consequentially, there is an increase in the capacity of novel sets in detecting marker-trait associations, even of alleles with minor or modest phenotypic effects (Risch and Zhang, 1996). In QTL linkage analysis, on the contrary, only a maximum of two alleles per locus are involved (diploid individual), with detection being restricted to the size of the effect and the presence of contrasts between genitor alleles in the desired trait.

As to grain quality traits, RM1 was associated to MR in the 2005 field experiment, and to yield QTL in previous works (Yu *et al.*, 1997; Brondani *et al.*, 2002; Septiningsih *et al.*, 2003), thereby indicating its location in a genomic region, and thus requiring detailed analysis, in order to identify genes and alleles of agronomic interest. In Brazil, rice breeders consider MR to be a very important trait, since cultivars, wherein the percentage of intact grains falls below 60%, are considered to be economically of less value. As to RM190 (*Waxy* gene), which is related to expression of the granule-bound starch-synthase enzyme, and is largely responsible for amylose content in rice grains (Ayres *et al.*, 1997), association to AC was reported in seven different segregating populations when applying QTL linkage analysis (He *et al.*, 1999; Tan *et al.*, 1999; Lanceras *et al.*, 2000; Septiningsih *et al.*, 2003; Zhou *et al.*, 2003; Aluko *et al.*, 2004; Fan *et al.*, 2005). AC was significantly related to RM190 through association analysis in both panels of upland and lowland accessions, throughout the two years.

The confirmation of an association between the RM190 marker and AC, when using core collection accessions, is additional evidence of efficiency in applying association analysis to gene identification. The significant results from Kruskal-Wallis testing, comprising experimental years and accession panels, implies that at least one of the alleles, exerted a discriminating effect on amylose content. Nevertheless, in spite of the lack of complete correspondence between RM190 alleles and amylose content, as to experimental-year and accession panel, some indication of a correlation for alleles 105 and 107 bp to intermediate amylose content, in the panel of lowland accessions and by experimental year, was found. On evaluating a panel of 89 non-waxy accessions, Ayres *et al.* (1997) identified seven alleles from a SSR marker in the *Wx* gene, whence four were correlated with different patterns of amylose content (high, intermediate and low), thus defining approximately 83% of AC variation. Bao *et al.* (2006) when examining a panel of 499 non-waxy accessions with RM190, identified ten alleles that together accounted for nearly 90% of AC variation. The difference of allelic correlation to amylose content classes recorded in the present work, Ayres *et al.* (1997) and Bao *et al.* (2006), could be due to the different composition of accession panels, since Ayres *et al.* (1997) analyzed a set of rice accessions from the U.S.A. with a narrower genetic base when compared to the ERiCC accession panel. Bao *et al.* (2006), when comparing the ERiCC accession panel, analyzed a panel of germplasm accessions with low diversity for the *Waxy* SSR marker, since from the ten alleles identified, only two accounted for a frequency of approximately 83%. The evaluation of germplasm accessions with a narrower genetic basis may be reflected in a low variation in modifier genes that could influence AC, particularly at the *Waxy* locus. This low variation in rice genomes, even in traits controlled by epistatic alleles, may restrict phenotypic trait variability. On the other hand, a wider genetic basis, as found in ERiCC accessions, could increase AC phenotypic variation, due to the allelic variability in genes located upstream of the *Waxy* gene in the starch synthesis route. This would reduce the power of a marker assisted selection in pre-breeding and breeding programs, if based solely on *Waxy* gene alleles, thereby requiring, as mandatory, the study of allelic variation in transcripts from the starch synthesis route in rice grains. As with Ayres *et al.* (1997), RM190 alleles with no relation to any specific class of amylose content were observed in the present work.

Starch makes up for 90% of polished grain in rice, and AC is recognized as one of the most important components in rice grain products (Bao *et al.*, 2006). The use of marker assisted selection for this trait would be of help in breeding programs, since accessions could be genotyped at an early stage for a trait that is normally evaluated after harvesting. However, the lack of correspondence in RM190 alleles to specific AC classes, as well as rice accessions with similar AC values, maybe showing differences in rice eating and textural qualities (Bao *et al.*, 2006), indicate the need for further studies before MAS implementation. Currently in the Embrapa breeding program, AC analysis is an annual, routine procedure in hundreds of inbred lines. In advanced elite inbred lines, the result of this analysis is being correlated with the direct determination of gel consistency and pasting viscosity by a panel of trained panelists, by using a small quantity of grains cooked in Petri dishes, thereby providing precise standards for defining grain quality.

This study demonstrated the feasibility of conducting association analysis together with germplasm characterization of a rice core collection using SSR markers. It also facilitated the identification of markers related to yield, panicle number, milled-head rice and amylose content, in a panel of genetically unrelated ERiCC accessions. Quantitative traits, such as yield, have complex gene and allele interactions, and studies to dissect this trait may start from QTL analysis, due to consolidated statistics and higher resolution potential. The expected high linkage disequilibrium of rice inbred lines and cultivars, although facilitating the detection of marker-trait associations, makes gene identification more difficult, since LD spans many thousands of base pairs. However, for breeding purposes the correlations detected by association analysis may be sufficient for marker assisted selection and mining alleles related to important traits in germplasm collections.

## Supplementary Material

The following online material is available for this article:

Table S1 Identification of ERiCC accessions, their origin and common name.

Table S2 Identification and information on SSR Markers used for ERiCC evaluation.

This material is made available as part of the online article from http://www.scielo.br.gmb.

## Figures and Tables

**Table 1:** Descriptive statistics for yield (YLD), tiller number (TILN), panicle number (PANN), yield from ratooning (RYLD), amylose content (AC) and head-milled rice (MR).

	2004 Experiment						2005 Experiment			
	YLD (kg/ha)	TILN	PANN	AC (%)	MR (%)		YLD (kg/ha)	RYLD (kg/ha)	AC (%)	MR (%)
Average	4685.0	166.43	143.3	24.3	60.4		4298.1	1403.4	24.1	43.1
Minimum	900	71	32	4	42.0		435	0	8	3.57
Maximum	8844	281	236	31	70.9		8130	3020	31	66.1
Standard deviation	41.6	6.7	6.2	2.5	2.5		39.0	24.4	2.2	3.8

BR IRGA 409^#^	5993.5	154	128	27	65.1		5372.3	1425.7	26	46.9
CAIAPO^#^	2948.7	114	102	26	-		5720.2	1125.0	24	61.9
METICA 1^#^	6243.5	197	175	25	-		3282.9	1884.2	25	43.17
COLOSSO^#^	3911.1	127	108	25	-		4785.0	1350.0	24	65.4

^#^ Controls of field experiments for both years.

**Table 2:** Pearson correlation coefficients among the phenotypic traits: yield (YLD), tiller number (TILN), panicle number (PANN), yield from ratooning (RYLD), amylose content (AC) and head-milled rice (MR).

		2004 Experiment						2005 Experiment			
		YLD (kg/ha)	TILN	PANN	AC (%)	MR (%)		YLD (kg/ha)	RYLD (kg/ha)	AC (%)	MR (%)
2004 Experiment	YLD	-									
	TILN	0.20**									
	PANN	0.31**	0.88**								
	AC	0.19**	0.26**	0.25**							
	MR	0.31**	-	-	-						

2005 Experiment	YLD	-	-	-	-	-					
	RYLD	-	-	-	-	-		-			
	AC	-	-	-	0.82**	-		0.20**	0.26**		
	MR	-	-	-	-	0.14*		0.38**	-	-	-

Only significant values are shown (*p < 0.05; **p < 0.01).

**Table 3:** Association of SSR markers with phenotypic traits. The statistics shown refer to the coefficient of determination (R^2^).

	Marker	Chromosome	Experiment 2004			Experiment 2005		
			PANN	AC		MR	YLD	AC
Lowland accessions	RM1	1	0.019	0.031		0.039* (*q*)	0.000	0.000
	RM38	8	0.083	0.049		0.021	0.040*(*q*)	0.074
	RM125	7	0.039	0.040		0.037	0.002*(*q*)	0.000
	RM190	6	0.062	0.425*(*q*)		0.012	0.049	0.36*(*q*)
	RM264	8	0.000	0.170		0.027	0.001*(*q*)	0.110
	RM267	5	0.020	0.019		0.020	0.011*(*q*)	0.035
	4653	12	0.137	0.068		0.004*(*q*)	0.143	0.070
	OG60	4	0.352*(*q*)	0.023		0.053	0.000	0.204
Upland accessions	RM190	6	0.000	0.390**(*q*)		0.000	0.090	0.490**(*q*)

Panicle number (PANN); amylose content (AC); head-milled rice (MR); yield (YLD). Only SSR markers with significant marker-trait association are given. The *q* indicates the false discovery rate control value set to 0.05. *p < 0.005; **p < 0.0001.

**Table 4:** Pairwise statistical differences in average amylose content values of each identified allele of the RM190 marker in both accession panels and over experimental years.

	105	107	117	119	121	125	PMR
105	-						
107	L/04	-					
117	U/05	U/05	-				
119	L/04, L/05	L/04, L/05	-	-			
121	L/04, U/04, U/05	L/04, L/05, U/04, U/05	-	-	-		
125	L/04	L/05	-	-	U/04, U/05	-	
PMR	-	-	-	-	U/04	-	-

L - Lowland accession panel. U - Upland accession panel. 04 - Data from 2004 experiment. 05 - Data from 2005 experiment. PMR - pool of missing and rare alleles for the RM190 marker.
